# Common patterns in the molecular phylogeography of western palearctic birds: a comprehensive review

**DOI:** 10.1007/s10336-021-01893-x

**Published:** 2021-05-13

**Authors:** Liviu G. Pârâu, Michael Wink

**Affiliations:** 1 Institute of Pharmacy and Molecular Biotechnology, Department Biology, Heidelberg University, Im Neuenheimer Feld 364, 4 OG, Heidelberg, Germany; 2 Present Address: SARS-CoV-2 Data Evaluation Office, Eurofins Genomics Europe Applied Genomics GmbH, Anzinger Straße 7a, 85560 Ebersberg, Germany

**Keywords:** Birds, Phylogeography, Western Palearctic, Europe, Genetic diversity, Panmixia, Pleistocene, Climate change

## Abstract

A plethora of studies have offered crucial insights in the phylogeographic status of Western Palearctic bird species. However, an overview integrating all this information and analyzing the combined results is still missing. In this study, we compiled all published peer-reviewed and grey literature available on the phylogeography of Western Palearctic bird species. Our literature review indicates a total number of 198 studies, with the overwhelming majority published as journal articles (*n* = 186). In total, these literature items offer information on 145 bird species. 85 of these species are characterized by low genetic differentiation, 46 species indicate genetic variation but no geographic structuring i.e. panmixia, while 14 species show geographically distinct lineages and haplotypes. Majority of bird species inhabiting the Western Palearctic display genetic admixture. The glaciation cycles in the past few million years were pivotal factors in shaping this situation: during warm periods many species expanded their distribution range to the north over wide areas of Eurasia; whereas, during ice ages most areas were no longer suitable and species retreated to refugia, where lineages mixed.

**Supplementary Information:**

The online version contains supplementary material available at 10.1007/s10336-021-01893-x.

## Abstract (de)

**Gemeinsame Muster in der molekularen Phylogeographie der westpaläarktischen Vögel: Ein umfassender Überblick**

Eine Fülle von genetischen Untersuchungen konnte entscheidende Einblicke in den phylogeographischen Status der westpaläarktischen Vogelarten geliefert. Es fehlt jedoch immer noch eine Übersicht, die all diese Informationen integriert und die Ergebnisse gemeinsam analysiert. In dieser Studie haben wir die gesamte veröffentlichte, begutachtete und graue Literatur zur Phylogeographie westpaläarktischer Vogelarten zusammengestellt. Unsere Literaturübersicht kommt auf eine Gesamtzahl von 198 Untersuchungen, wobei die überwiegende Mehrheit als Zeitschriftenartikel veröffentlicht wurde (*n* = 186). Insgesamt bieten diese Publikationen Informationen zu 145 Vogelarten. 85 dieser Arten zeichnen sich durch eine geringe genetische Differenzierung aus, 46 Arten zeigen genetische Variation, aber keine geografische Strukturierung, d. h. Panmixie, während nur14 Arten geografisch unterschiedliche Linien und Haplotypen aufweisen. Die Mehrheit der Vogelarten, die die Westpaläarktis besiedeln, zeigen eine genetische Vermischung. Die Vergletscherungszyklen der letzten paar Millionen Jahre waren ausschlaggebend für diese Situation: Während Warmzeiten dehnten viele Arten ihr Verbreitungsgebiet über weite Teile Eurasiens nach Norden aus; während der Eiszeiten waren die meisten Gebiete zur Besiedlung nicht mehr geeignet und die Arten zogen sich in Refugien zurück. Diese klimatischen Zyklen und periodischen Verbreitungsänderungen innerhalb der letzten zwei Millionen Jahre führten zur Durchmischung der genetischen Entwicklungslinien, wie wir sie heute feststellen können.

## Introduction

Past climatic events are embedded in the DNA of organisms (Hewitt 2000). Across the paleogeographic areas of the world, local fauna shares molecular evidence indicating how they responded to the major successions of cold and warm periods (Webb and Bartlein 1992). In the past three decades, the emergence of fine-tuned molecular techniques triggered a *renaissance* in zoology. Based on DNA sequencing, scientists were finally able to decipher the chronological and spatial evolution of species and subspecies (Hewitt 1999). With the possibility of assessing the progress of a species in both time and space, the field of phylogeography emerged (Avise et al. 1987). This served as an unprecedented opportunity and soon after the first studies began to appear (Cwynar and MacDonald 1987; Martin and Simon 1990; Reeb and Avise 1990; Nevo and Beiles 1992; Prinsloo and Robinson 1992; Quinn 1992; Melnick et al. 1993). Initial studies had a limited focus, dealing with small sample sizes and generally employed a single molecular marker, most often a mitochondrial DNA (hereafter mtDNA) gene. Moreover, in the first few years, both software and statistical techniques were a crude limitation to data analysis (Edwards and Bensch 2009).

As advanced DNA sequencing techniques became widely available, phylogeography became more popular, and the field entered a period of formidable growth (Hickerson et al. 2010), with thousands of studies currently being available. One would expect that a considerable amount of work would also have been done in bringing together and interpreting this colossal volume of data. However, such reviews are very scarce, and they generally have a wide focus, from assessing the hypotheses behind the genetic lineages, to number of papers and species. To date, review articles have included the status of phylogeographic research for oceanic habitats at global level (Bowen et al. 2016), or for archipelagos (Shaw and Gillespie 2016). Reviews are also available for research on continental areas (Riddle 2016), or studies dealing exclusively with the Southern hemisphere (Beheregaray 2008). One review examined exclusively the terrestrial taxa in the Aegean archipelago and surrounding regions (Poulakakis et al. 2015). Among all the vertebrates which became a research focus in phylogeography, birds proved to be the most iconic (Weiss and Ferrand 2007).

Numerous avian species had their phylogeography revealed. In Eurasia, the bird families which received most attention are the raptors, especially the Accipitridae, the crows (Corvidae), flycatchers and wheatears (both in Muscicapidae), as well as species from Phasianidae and the waders (Scolopacidae). To date, surprisingly few review and comparative studies have focused on birds. The first, authored by Robert M. Zink (Zink 1996), analyzed mtDNA geographic patterns of five North American bird species, to determine that their absence of genetic structure is linked to recent population expansion. The second, a book section in 1997, written by the same author (Zink 1997), offers an improved version of the first review, with more species included, which overall indicates the same population genetic structure. The third, in 1998, written by John C. Avise and DeEtte Walker (Avise and Walker 1998), compares 63 species from a phylogeographic and speciation perspective, confirming that the Pleistocene had a decisive effect on avian speciation. The fourth, as a chapter in the PhD thesis of Alexandra Pavlova (Pavlova 2004), has compared phylogeographic information of 28 Eurasian bird species, concluding that the South contains regions with higher genetic diversity and phylogeographic endemism, with the overall genetic structure being shaped by the post-Pleistocene recolonization of Eurasia. In 2009, a review focused on the phylogeography of birds from the Australo-Papuan region, indicated extensive paraphyly among birds in Australia (Joseph and Omland 2009). Following the chronological order, the next review brings into attention the horizontal and elevational phylogeographical patterns of Himalayan and Southeast Asian birds (Päckert et al. 2012). The seventh study collected phylogeographic data for 210 bird species in the New World, to reveal that species from lower latitudes have higher genetic diversity (Smith et al. 2017). The most recent study, authored by Alexey P. Kryukov (Kryukov 2019) has an exclusive focus on Palearctic corvid species and reviews published data on the natural hybrid zones between crow species.

To the best of our knowledge, no study aimed specifically at deciphering the avian phylogeography of the Western Palearctic has been published. This region has received extensive attention, triggered both by the geographic composition e.g. various islands promoting endemism, as well as the density of research groups and availability of funding. In this study, we compiled a comprehensive body of published scientific literature with a clear focus on phylogeography of bird species inhabiting the Western Palearctic.

### Survey methodology

Between November 2018 and June 2020, we conducted an extensive search for relevant literature on: (1) ISI Web of knowledge, (2) Google Scholar, (3) Research Gate and (4) Google. We used the following keywords: “phylogeography” AND (“bird” OR “avian”) AND (“Western Palearctic” OR “Europe”). Our initial searches were made using English, French, Spanish, German and Russian, which are recognized as some of the most used languages for scientific publishing in our target region (Ammon 2001; Ammon and McConnell 2002). However, as we only found proper results in English, we excluded the other languages from further searches. Moreover, we considered both peer-reviewed and grey literature. We extracted additional papers from the references of the articles revealed by our systematic review, when the title or citation context indicated a bird phylogeography investigation. This study is limited to the area of the Western Palearctic, as presented in Shirihai & Svensson (Shirihai and Svensson 2018), consisting of Europe, North Africa, the Middle East and Asia Minor, the Cape Verde, Azores and Canary Islands, Madeira, Jan Mayen, Svalbard and Iceland. As the Eastern limit, we expanded until the Caspian Sea and the Ural Mountains. For taxonomy, we followed the IOC World Bird List (Gill et al. 2020).

The papers revealed by our on-line searches were further filtered according to the following criteria: (i) to focus on naturally occurring species in the Western Palearctic and (ii) to have samples originating from at least three geographically distinct populations. These facts were obtained upon reading the abstract plus materials and methods section. We later extracted information on the phylogeographic status of the studied species and assigned it one of the three categories: (i) panmixia, when the haplotypes are randomly distributed across the sampled area and no structure can be observed; (ii) low differentiation, if a certain degree of geographic delimitation of the haplotypes occur yet the lineage sorting is incomplete (e.g. Western vs. Eastern European haploclades, continental Europe vs. UK lineages) or (iii) geographically distinct lineages, for cases where certain haplotypes can be safely attributed to a geographic area and monophyletic groups are present (e.g. haplotypes found exclusively in one mountain range).

In addition, for each bird species, we noted the type of molecular technique (markers) used for obtaining the data and the migratory status of the respective species. The later information was retrieved from the IUCN Red List (IUCN 2019). We used the program R (R Core Team 2019) for data visualization.

To offer a better visualization of the phylogeographic differences among the three defined categories, we selected one species for each category, constructed its haplotype network and positioned all three networks side by side. We chose the European Turtle Dove (*Streptopelia turtur*) to illustrate panmixia, the European Green Woodpecker (*Picus viridis*) for low geographic differentiation and the African Blue Tit (*Cyanistes teneriffae*) as model for the geographically distinct lineages category. Complete details on the GenBank sequences used for the visualization are found in Table S4. Mainly, the sequences are derived from the following studies: (Calderón et al. 2016) for the dove, (Perktas et al. 2011) for the woodpecker and (Dietzen et al. 2008) for the tit. After downloading the sequences from GenBank, we grouped all files belonging to one species into a fasta file, using MEGA X (Kumar et al. 2018). We further assessed the number of haplotypes in DNA SP (Rozas et al. 2017) and finally employed the PopArt software (Leigh and Bryant 2015) to create the networks.

## Results

In total, 145 bird species from the Western Palearctic have been the target of phylogeographic studies (Tables 1, 2, 3, Table S1). A number of 198 literature items (Table S2), including 186 peer-reviewed articles, one preprint, four PhD theses and one Master thesis, four articles in conference proceedings, and two book chapters matched our literature selection criteria. The year of publication ranges from 1993 until 2020. The Western Capercaillie (*Tetrao urogallus)* has been the focus of ten publications, which makes it the most investigated species in our dataset.

**Table 1:** Western Palearctic bird species which show geographically distinct lineages (*n* = 14)

Nr	Scientific name	English name	Migratory status	Molecular marker	Reference
1	*Aegypius monachus*	Cinereous Vulture	Resident	Msats & mtDNA	(Poulakakis et al. 2008; Çakmak et al. 2019)
2	*Alectoris graeca*	Rock Partridge	Resident	Msats & mtDNA	(Lucchini and Randi 1998; Randi et al. 2003)
3	*Cyanistes teneriffae*	African Blue Tit	Resident	NuDNA, msats, mtDNA & NGS	(Kvist et al. 2005; Dietzen et al. 2008; Illera et al. 2011; Stervander et al. 2015)
4	*Cyanopica cooki*	Iberian Magpie	Resident	MtDNA	(Haring et al. 2007)
5	*Fringilla polatzeki*	Gran Canaria Blue Chaffinch	Resident	NuDNA & mtDNA	(Pestano et al. 2000; Garcia-del-Rey et al. 2013; Lifjeld et al. 2016)
6	*Fringilla teydea*	Tenerife Blue Chaffinch	Resident	NuDNA & mtDNA	(Pestano et al. 2000; Garcia-del-Rey et al. 2013; Lifjeld et al. 2016)
7	*Galerida cristata*	Crested Lark	Partial migrant	MtDNA	(Guillaumet et al. 2006)
8	*Hydrobates pelagicus*	European Storm Petrel	Resident	MtDNA	(Cagnon et al. 2004)
9	*Phylloscopus canariensis*	Canary Islands Chiffchaff	Resident	MtDNA	(Illera et al. 2020)
10	*Serinus canaria*	Atlantic Canary	Resident	MtDNA	(Dietzen et al. 2006)
11	*Sitta krueperi*	Krüper's Nuthatch	Resident	Msats & mtDNA	(Albayrak et al. 2012)
12	*Somateria mollissima*	Common Eider	Migrant	Msats & mtDNA	(Tiedemann et al. 2004)
13	*Strix aluco*	Tawny Owl	Resident	MtDNA	(Brito 2005)
14	*Tetrastes bonasia*	Hazel Grouse	Resident	Msats & mtDNA	(Sahlsten et al. 2008; Rutkowski et al. 2012, 2016)

**Table 2:** Western Palearctic bird species which show panmixia (*n* = 46)

Nr	Scientific name	English name	Migratory status	Molecular marker	Reference
1	*Accipiter gentilis*	Northern goshawk	Resident	MtDNA	(Kunz et al. 2019)
2	*Acrocephalus palustris*	Marsh warbler	Migrant	MtDNA	(Arbabi et al. 2014a)
3	*Actitis hypoleucos*	Common sandpiper	Migrant	MtDNA	(Zink et al. 2008)
4	*Bubo scandiacus*	Snowy owl	Partial migrant	Sex chromosomes & mtDNA	(Marthinsen et al. 2009)
5	*Buteo buteo*	Common buzzard	Partial migrant	NuDNA, msats & mtDNA	(Jowers et al. 2019)
6	*Buteo rufinus*	Long-legged buzzard	Partial migrant	NuDNA, msats & mtDNA	(Jowers et al. 2019)
7	*Calidris canutus*	Red knot	Migrant	MtDNA	(Baker et al. 1994)
8	*Calidris pugnax*	Ruff	Migrant	NuDNA & mtDNA	(Verkuil et al. 2012)
9	*Columba palumbus*	Common wood pigeon	Partial migrant	MtDNA	(Grosso et al. 2006)
10	*Corvus corax*	Northern raven	Resident	MtDNA	(Haring et al. 2007; Rösner et al. 2014)
11	*Dendrocopos major*	Great spotted woodpecker	Resident	MtDNA	(Zink et al. 2002a; Garcia-del-Rey et al. 2007; Perktas and Quintero 2013)
12	*Emberiza sahari*	House bunting	Resident	MtDNA	(Schweizer et al. 2018)
13	*Falco cherrug*	Saker falcon	Migrant	NuDNA, msats & mtDNA	(Nittinger et al. 2007; Zhan et al. 2015)
14	*Ficedula albicollis*	Collared flycatcher	Migrant	NuDNA	(Backström et al. 2013)
15	*Grus grus*	Common crane	Migrant	Msats	(Haase et al. 2019)
16	*Haliaeetus albicilla*	White-tailed eagle	Partial migrant	Msats & mtDNA	(Honnen et al. 2010; Langguth et al. 2013; Nemesházi et al. 2016)
17	*Hieraaetus fasciatus*	Bonelli's eagle	Resident	MtDNA	(Cardia et al. 2000; Cadahía et al. 2007)
18	*Hirundo rustica*	Barn swallow	Migrant	NGS, msats & mtDNA	(von Rönn et al. 2016)
19	*Lanius collurio*	Red-backed shrike	Migrant	MtDNA	(Pârâu et al. 2019)
20	*Lanius minor*	Lesser grey shrike	Migrant	NuDNA & mtDNA	(Kvist et al. 2011)
21	*Locustella luscinioides*	Savi's warbler	Migrant	Msats & mtDNA	(Neto et al. 2012)
22	*Loxia curvirostra*	Red crossbill	Partial migrant	MtDNA	(Questiau et al. 1999)
23	*Luscinia svecica*	Bluethroat	Migrant	MtDNA	(Zink et al. 2003)
24	*Mareca penelope*	Eurasian wigeon	Migrant	MtDNA	(Kulikova et al. 2019)
25	*Merops apiaster*	European bee-eater	Migrant	Msats & mtDNA	(de Melo et al. 2019)
26	*Motacilla alba*	White wagtail	Migrant	NGS, nuDNA & mtDNA	(Pavlova et al. 2005b; Li et al. 2016; Harris et al. 2018)
27	*Motacilla cinerea*	Grey wagtail	Migrant	NGS, nuDNA & mtDNA	(Harris et al. 2018)
28	*Motacilla citreola*	Citrine wagtail	Migrant	NGS, nuDNA & mtDNA	(Pavlova et al. 2003; Harris et al. 2018)
29	*Motacilla flava*	Yellow wagtail	Migrant	NGS, nuDNA & mtDNA	(Pavlova et al. 2003; Harris et al. 2018)
30	*Muscicapa striata*	Spotted flycatcher	Migrant	NuDNA & MtDNA	(Pons et al. 2016)
31	*Netta rufina*	Red-crested pochard	Migrant	Msats & mtDNA	(Gay et al. 2004)
32	*Nucifraga caryocatactes*	Spotted nutcracker	Resident	MtDNA	(Haring et al. 2007; Dohms and Burg 2014; Dohms 2016)
33	*Numenius phaeopus*	Eurasian whimbrel	Migrant	NGS	(Tan et al. 2019)
34	*Pandion haliaetus*	Osprey	Migrant	MtDNA	(Monti et al. 2015)
35	*Parus major*	Great tit	Partial migrant	NGS, nuDNA & mtDNA	(Kvist et al. 1999; Kvist 2000; Pavlova et al. 2006; Spurgin et al. 2019; Song et al. 2020)
36	*Perisoreus infaustus*	Siberian jay	Resident	MtDNA	(Haring et al. 2007)
37	*Phoenicurus phoenicurus*	Common redstart	Migrant	MtDNA	(Hogner et al. 2012)
38	*Phylloscopus trochilus*	Willow warbler	Migrant	Msats & mtDNA	(Bensch et al. 1999)
39	*Picoides tridactylus*	Eurasian three-toed woodpecker	Resident	MtDNA	(Zink et al. 2002b)
40	*Poecile montanus*	Willow tit	Partial migrant	MtDNA	(Kvist et al. 2001; Salzburger et al. 2002; Pavlova et al. 2006)
41	*Streptopelia decaocto*	Eurasian collared dove	Resident	MtDNA	(Bagi et al. 2018)
42	*Streptopelia turtur*	European turtle dove	Migrant	NGS & mtdna	(Calderón et al. 2016)
43	*Sylvia atricapilla*	Eurasian blackcap	Migrant	MtDNA	(Perez-Tris et al. 2004)
44	*Tetrax tetrax*	Little bustard	Migrant	MtDNA	(Garcia et al. 2011)
45	*Upupa epops*	Eurasian hoopoe	Migrant	Msats & mtDNA	(Wang et al. 2017)
46	*Xenus cinereus*	Terek sandpiper	Migrant	Msats & mtDNA	(Rönkä et al. 2019)

**Table 3:** Western Palearctic bird species which show low geographic differentiation (*n* = 85)

Nr	Scientific name	English name	Migratory status	Molecular marker	Reference
1	*Acrocephalus agricola*	Paddyfield warbler	Migrant	Msats & mtDNA	(Zehtindjiev et al. 2011)
2	*Acrocephalus arundinaceus*	Great reed warbler	Migrant	MtDNA	(Bensch and Hasselquist 1999; Hansson et al. 2008)
3	*Acrocephalus scirpaceus*	Eurasian reed warbler	Migrant	MtDNA	(Arbabi et al. 2014b; Olsson et al. 2016)
4	*Aegithalos caudatus*	Long-tailed tit	Partial migrant	NuDNA & MtDNA	(Zink et al. 2008; Song et al. 2016)
5	*Aegolius funereus*	Boreal owl	Resident	Msats	(Koopman et al. 2005)
6	*Alauda arvensis*	Eurasian skylark	Migrant	MtDNA	(Zink et al. 2008)
7	*Alaudala rufescens*	Lesser short-toed lark	Migrant	NuDNA & mtDNA	(Ghorbani et al. 2020b)
8	*Alectoris rufa*	Red-legged partridge	Resident	Msats & mtDNA	(Barbanera et al. 2011)
9	*Anas platyrhynchos*	Mallard	Partial migrant	MtDNA	(Hou et al. 2011; Kulikova et al. 2012)
10	*Anser brachyrhynchus*	Pink-footed goose	Migrant	MtDNA	(Ruokonen et al. 2005)
11	*Aquila adalberti*	Spanish imperial eagle	Resident	Msats & mtDNA	(Martinez-Cruz et al. 2004)
12	*Aquila chrysaetos*	Golden eagle	Partial migrant	Msats & mtDNA	(Nebel et al. 2015, 2019)
13	*Aquila heliaca*	Eastern imperial eagle	Migrant	Msats & mtDNA	(Vili et al. 2009; Korepov et al. 2017)
14	*Arenaria interpres*	Ruddy turnstone	Migrant	MtDNA	(Wenink et al. 1994)
15	*Athene noctua*	Little owl	Resident	Msats & mtDNA	(Wink 2008; Pellegrino et al. 2014, 2015)
16	*Burhinus oedicnemus*	Eurasian stone-curlew	Partial migrant	Msats & mtDNA	(Mori et al. 2014, 2017)
17	*Calidris alpina*	Dunlin	Migrant	Sex chromosomes, msats & mtDNA	(Wenink et al. 1993, 1996; Marthinsen et al. 2007; Wennerberg et al. 2008; Lopes et al. 2008)
18	*Calidris maritima*	Purple sandpiper	Migrant	Msats & mtDNA	(LeBlanc et al. 2017)
19	*Calonectris diomedea*	Scopoli's shearwater	Migrant	MtDNA	(Gómez-Díaz et al. 2006)
20	*Calonectris edwardsii*	Cape Verde shearwater	Migrant	MtDNA	Ibid
21	*Carduelis citrinella*	Citril finch	Migrant	Msats & mtDNA	(Pasquet and Thibault 1997; Senar et al. 2006)
22	*Carpodacus erythrinus*	Common rosefinch	Migrant	Sex chromosomes & mtDNA	(Pavlova et al. 2005a; Hung et al. 2012b)
23	*Certhia familiaris*	Eurasian treecreeper	Partial migrant	NuDNA, msats & mtDNA	(Pons et al. 2015, 2019a)
24	*Charadrius hiaticula*	Common ringed plover	Migrant	Msats	(Thies et al. 2018)
25	*Cinclus cinclus*	White-throated dipper	Partial migrant	Msats & mtDNA	(Lauga et al. 2005; Hourlay et al. 2008; Hernández et al. 2012, 2016)
26	*Clanga clanga*	Great spotted eagle	Migrant	MtDNA	(Väli 2004; Väli et al. 2004)
27	*Clanga pomarina*	Lesser spotted eagle	Migrant	MtDNA	Ibid
28	*Corvus corone*	Carrion crow	Resident	MtDNA	(Haring et al. 2007)
29	*Corvus frugilegus*	Rook	Resident	MtDNA	Ibid
30	*Corvus monedula*	Jackdaw	Resident	MtDNA	Ibid
31	*Cyanistes caeruleus*	Eurasian blue tit	Partial migrant	NuDNA, msats, mtDNA & NGS	(Kvist et al. 1999, 2004; Kvist 2000; Illera et al. 2011; Stervander et al. 2015)
32	*Cyanistes cyanus*	Azure tit	Partial migrant	NuDNA, msats, mtDNA & NGS	(Illera et al. 2011; Stervander et al. 2015)
33	*Dendrocoptes medius*	Middle spotted woodpecker	Resident	Sex chromosomes & mtDNA	(Kamp et al. 2018)
34	*Emberiza hortulana*	Ortolan bunting	Migrant	NGS & msats	(Moussy et al. 2018)
35	*Emberiza schoeniclus*	Common reed bunting	Migrant	MtDNA	(Zink et al. 2008)
36	*Eremophila alpestris*	Horned lark	Migrant	Sex chromosomes, nuDNA & mtDNA	(Drovetski et al. 2014; Ghorbani et al. 2020a)
37	*Eremophila bilopha*	Temminck's lark	Resident	mtDNA	(Ghorbani et al. 2020a)
38	*Erithacus rubecula*	European robin	Partial migrant	NuDNA & mtDNA	(Dietzen et al. 2003; Rodrigues et al. 2013)
39	*Falco naumanni*	Lesser kestrel	Migrant	MtDNA	(Wink et al. 2004)
40	*Falco peregrinus*	Peregrine falcon	Migrant	MtDNA	(Wink 2018b)
41	*Ficedula hypoleuca*	European pied flycatcher	Migrant	Msats & nuDNA	(Lehtonen et al. 2009; Backström et al. 2013)
42	*Ficedula parva*	Red-breasted flycatcher	Migrant	MtDNA	(Zink et al. 2008)
43	*Fringilla coelebs*	Common chaffinch	Partial migrant	NuDNA & mtDNA	(Suárez et al. 2009; Rodrigues et al. 2014b; Illera et al. 2018)
44	*Gypaetus barbatus*	Bearded vulture	Resident	MtDNA	(Godoy et al. 2004)
45	*Gyps fulvus*	Griffon vulture	Resident	Msats	(Arshad et al. 2009)
46	*Lagopus lagopus*	Willow ptarmigan	Resident	MtDNA	(Lagerholm et al. 2017)
47	*Lagopus muta*	Rock ptarmigan	Resident	MtDNA	Ibid
48	*Lanius meridionalis*	Iberian grey shrike	Resident	NuDNA & mtDNA	(Gonzalez et al. 2008)
49	*Lanius meridionalis koenigi*	Southern grey shrike	Resident	Msats & mtDNA	(Padilla et al. 2015)
50	*Larus argentatus*	European herring gull	Partial migrant	NuDNA, msats & mtDNA	(Sonsthagen et al. 2012)
51	*Larus armenicus*	Armenian gull	Partial migrant	MtDNA	(Liebers et al. 2001)
52	*Larus fuscus*	Lesser black-backed gull	Partial migrant	MtDNA	(Liebers and Helbig 2002)
53	*Larus michahellis*	Yellowed-legged gull	Partial migrant	MtDNA	(Liebers et al. 2001)
54	*Limosa limosa*	Black-tailed godwit	Migrant	MtDNA	(Höglund et al. 2009)
55	*Lyrurus tetrix*	Black grouse	Resident	Msats & mtDNA	(Höglund et al. 2007; Corrales et al. 2014; Sittenthaler et al. 2018; Rutkowski et al. 2019)
56	*Milvus milvus*	Red kite	Partial migrant	MtDNA	(Roques and Negro 2005)
57	*Montifringilla nivalis*	White-winged snowfinch	Partial migrant	Hydrogen isotopes and mtDNA	(Resano-Mayor et al. 2017)
58	*Numenius arquata*	Eurasian curlew	Migrant	NuDNA & mtDNA	(Tan et al. 2019; Rodrigues et al. 2019)
59	*Oenanthe cypriaca*	Cyprus wheatear	Migrant	NuDNA & mtDNA	(Randler et al. 2012; Alaei Kakhki et al. 2018)
60	*Oenanthe hispanica*	Black-eared wheatear	Migrant	NuDNA & mtDNA	(Alaei Kakhki et al. 2018)
61	*Oenanthe oenanthe*	Northern wheatear	Migrant	NGS & mtDNA	(Wang et al. 2018, 2020)
62	*Oenanthe pleschanka*	Pied wheatear	Migrant	NuDNA & mtDNA	(Alaei Kakhki et al. 2018)
63	*Otis tarda*	Great bustard	Resident	NuDNA & mtDNA	(Pitra et al. 2000)
64	*Perdix perdix*	Grey partridge	Resident	MtDNA	(Liukkonen-Anttila et al. 2002)
65	*Periparus ater*	Coal tit	Resident	Msats & mtDNA	(Pentzold et al. 2013; Tritsch et al. 2018)
66	*Phalacrocorax carbo*	Great cormorant	Partial migrant	MtDNA	(Winney et al. 2001; Marion and Le Gentil 2006)
67	*Phylloscopus collybita*	Common chiffchaff	Migrant	MtDNA	(Raković et al. 2019)
68	*Pica pica*	Eurasian magpie	Resident	NuDNA & mtDNA	(Haring et al. 2007; Kryukov et al. 2017; Song et al. 2018)
69	*Picus viridis*	European green woodpecker	Resident	Sex chromosomes, nuDNA & MtDNA	(Perktas et al. 2011; Pons et al. 2011, 2019b)
70	*Prunella modularis*	Dunnock	Migrant	MtDNA	(Drovetski et al. 2018)
71	*Regulus regulus*	Goldcrest	Migrant	NuDNA & mtDNA	(Rodrigues et al. 2014a)
72	*Riparia riparia*	Sand martin	Migrant	NuDNA & mtDNA	(Pavlova et al. 2008)
73	*Sitta europea*	Eurasian nuthatch	Resident	NuDNA & mtDNA	(Zink et al. 2006; Hung et al. 2012a; Päckert et al. 2020)
74	*Sitta neumayer*	Western rock nuthatch	Partial migrant	MtDNA	(Elverici 2018)
75	*Sitta tephronota*	Eastern rock nuthatch	Partial migrant	MtDNA	Ibid
76	*Sitta whiteheadi*	Corsican nuthatch	Resident	NuDNA & mtDNA	(Thibault et al. 2016)
77	*Strix uralensis*	Ural owl	Resident	NuDNA & mtDNA	(Hausknecht et al. 2014)
78	*Sylvia cantillans*	Subalpine warbler	Migrant	NuDNA & mtDNA	(Brambilla et al. 2008; Zuccon et al. 2020)
79	*Sylvia conspicillata*	Spectacled warbler	Partial migrant	Msats & mtDNA	(Illera et al. 2014)
80	*Sylvia curruca*	Lesser whitethroat	Migrant	NuDNA & mtDNA	(Olsson et al. 2013; Abdilzadeh et al. 2020)
81	*Sylvia subalpina*	Moltoni's warbler	Migrant	NuDNA & mtDNA	(Brambilla et al. 2008; Zuccon et al. 2020)
82	*Tetrao urogallus*	Western capercaillie	Resident	Msats & mtDNA	(Segelbacher and Storch 2002; Segelbacher et al. 2003; Liukkonen-Anttila et al. 2004; Rutkowski et al. 2005, 2017; Duriez et al. 2007; Rodríguez-Muñoz et al. 2007; Segelbacher and Piertney 2007; Bajc et al. 2011; Klinga et al. 2020)
83	*Tringa totanus*	Common redshank	Migrant	NuDNA & mtDNA	(Ottvall et al. 2005)
84	*Troglodytes troglodytes*	Eurasian wren	Partial migrant	MtDNA	(Drovetski et al. 2004; Shannon et al. 2014; Albrecht et al. 2018, 2020)
85	*Turdus merula*	Common blackbird	Partial migrant	Sex chromosomes & mtDNA	(Rodrigues et al. 2016)

**Fig. 1:**
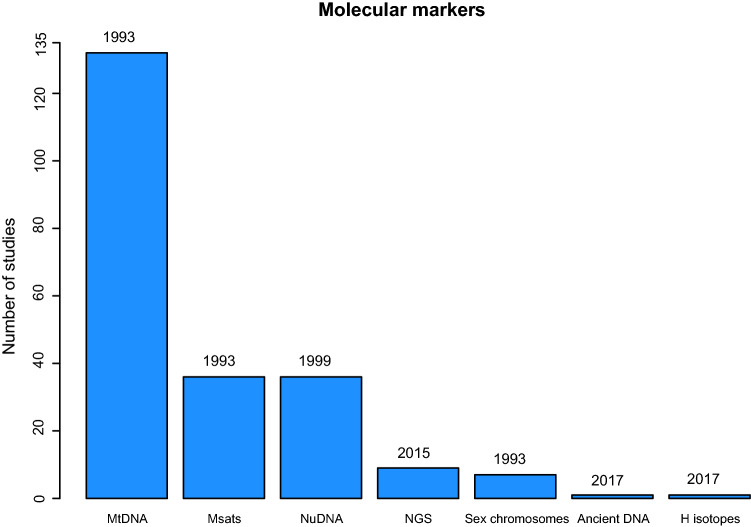
Molecular markers used in the studies identified in our literature review: mitochondrial DNA, in 132 studies, microsatellites—36, nuclear DNA—36, NGS—7, sex chromosomes—7, ancient DNA—1, hydrogen isotopes—1. Year on top of each bar represents the first use of the respective marker

In terms of molecular markers, for the majority of bird species (132 out of 145) the choice has been mtDNA (Fig. 1), which is also one of the three markers used since the first studies in 1993. Secondly, microsatellites have been used for 36 species and nuclear DNA sequences (hereafter nuDNA) for 36 as well. Seven studies have employed sex chromosomes and a further seven studies used next-generation DNA sequencing (hereafter NGS). In the NGS category we included research with whole-genome sequencing, ddRAD sequencing and SNPs. One study (Resano-Mayor et al. 2017) has used hydrogen isotopes in combination with mtDNA. Furthermore, one study (Lagerholm et al. 2017) employed ancient DNA extracted from fossil bones. For each marker, the above-mentioned values consist of both the occasions where the respective marker has been applied alone or in combination with another marker.

**Fig. 2:**
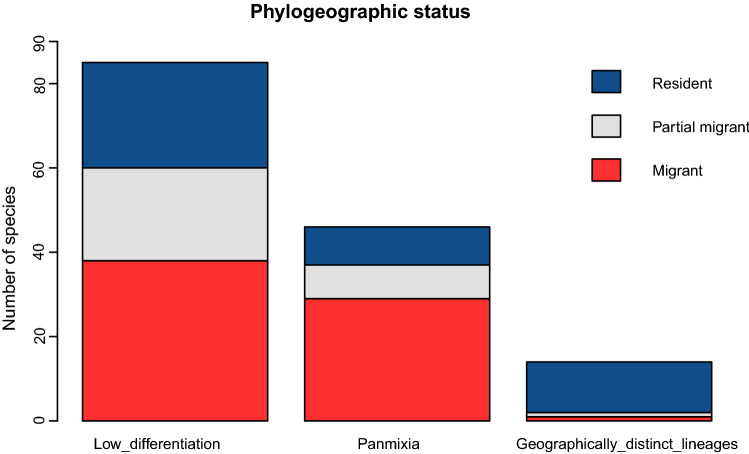
Phylogeographic status of the 145 bird species in our study: low differentiation—85, panmixia—46, geographically distinct lineages—14. In addition, each bar indicates the migratory behavior of the comprising species

Across the Western Palearctic, 85 avian species show signs of low genetic differentiation (Fig. 2), while 46 are genetically diverse but do not show a geographic structuring—indicating panmixia, and the remaining 14 species display geographically distinct lineages. Regarding the migratory behavior of the species comprising each category, we observed that the majority of the birds with low genetic differentiation and panmixia are migratory, while the species showing geographically distinct lineages are mainly resident and/or inhabitants of Oceanic islands (Fig. 2).

**Fig. 3:**
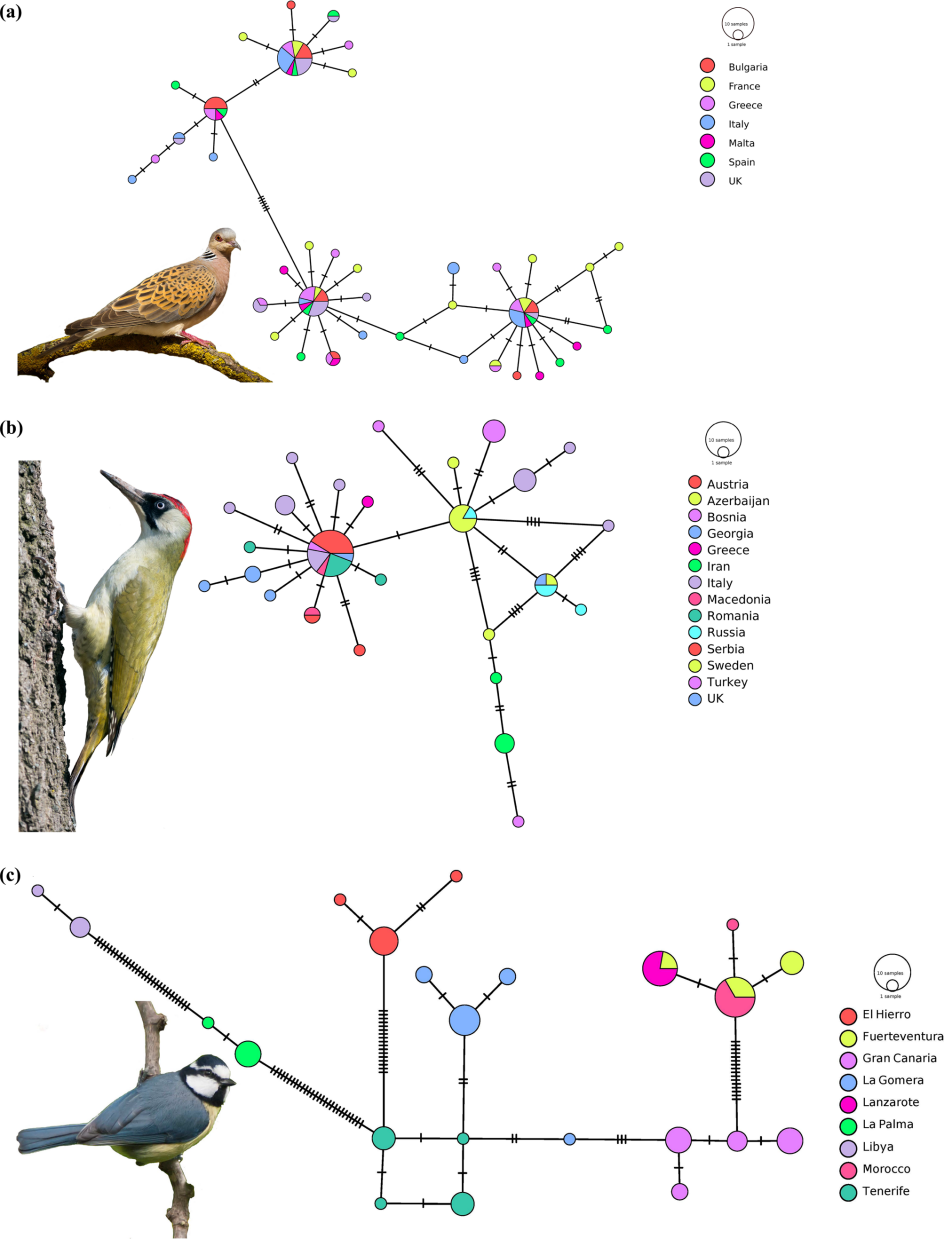
Comparative haplotype networks for the three species of birds, representative for each phylogeographic category: **a** European Turtle Dove (*Streptopelia turtur*) indicating panmixia, **b** European Green Woodpecker (*Picus viridis*) showing low geographic differentiation and **c** African Blue Tit (*Cyanistes teneriffae)* indicating geographically distinct lineages. The sequences are derived from the following studies: Calderón et al. 2016 (Calderón et al. 2016) for the dove (Cytb sequences), Perktaș et al. 2011 (Perktas et al. 2011) for the woodpecker (ND2 sequences) and Dietzen et al. 2008 (Dietzen et al. 2008) for the tit (Cytb sequences). This figure is available in higher quality as Figure S3

Our haplotype network comparison revealed substantial differences among the three selected species. To visualize the situation for birds with panmixia, we chose the European Turtle Dove (*Streptopelia turtur*). For species with low geographic differentiation, we selected the European Green Woodpecker (*Picus viridis*). We chose the African Blue Tit (*Cyanistes teneriffae*), as a model for the geographically distinct lineages category. Figure 3 (and in higher quality as figure S3) contains our visual comparison, which points to the differences in the distribution of haplotypes, among the three species. For the European Turtle Dove, all seven countries share haplotypes, regardless of the distance in between e.g. UK and Bulgaria. The network for the European Green Woodpecker indicates that several haplotypes are shared among the various populations, but some countries have specific haplotypes e.g. Italy, Turkey, Greece. It should be mentioned, that the Iberian population of the Green Woodpecker was found to be genetically different and has been consequently split as a new species *Picus sharpei* (Perktas et al. 2011; Pons et al. 2011). For the African Blue Tit, which is found in the Canary Islands, plus in Morocco, Algeria, Tunisia and Libya, the haplotype distribution is very clear. Majority of the islands in the Canary archipelago have distinct haplotypes, which are not found on the other islands. On the African continent, Libya has its own haplotypes, while Moroccan birds appear to be sharing some genetic background with birds from Fuerteventura. Complete details on the GenBank sequences used for the visualization are found in Table S4.

## Discussion

### Phylogeographical situation of Western Palearctic birds

**Fig. 4:**
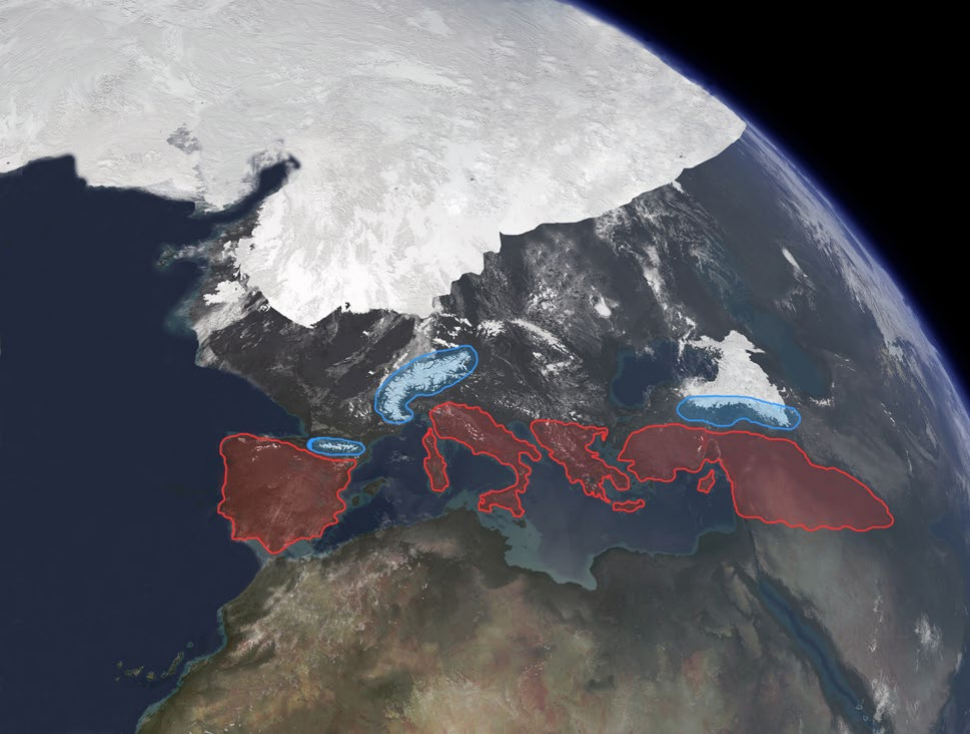
Visualization of the Western Palearctic during glaciation, with blue areas depicting possible alpine refugia, while the red ones indicate Southern refugia

Substantial research has been carried out to understand the phylogeographic history of Western Palearctic avifauna, resulting in 145 bird species being studied. This represents roughly 20% of the 720 bird species found in the region (Shirihai and Svensson 2018). Our review indicates that majority of the species are characterized by similar patterns of genetic variation and admixture. This situation is inextricably connected to the climatic past of the Western Palearctic (Lisiecki and Raymo 2007), with the evidence being encrypted in the DNA of the species inhabiting this region (Taberlet et al. 1998; Weiss and Ferrand 2007). The periods mainly responsible for shaping the genetic background for the current avifauna are the Pleistocene and Pliocene (Rand 1948; Avise and Walker 1998; Zink et al. 2004). During these two eras, the climate oscillated between glaciations and warm cycles, accompanied by shifts in the composition of the vegetation (Frenzel et al. 1992). In turn, these oscillations led to massive bird population crashes at the arrival of each glaciation, or great population expansions, when the ice sheet retreated (Hewitt 1999). Subsequently, these demographic processes triggered population admixture both in the refugia (see Fig. 4) and at the contact zones (during population expansion times). As a result, the overwhelming majority of bird species in our dataset (i.e. 131 out of 145, or 90.3%) are characterized by high levels of genetic admixture, with either complete panmixia or low differentiation among various breeding populations.

Furthermore, the majority (i.e. 97 out of 131, or 74%) of species with genetic admixture are migrants or partial migrants (see Fig. 2). This suggests that the legacy of migration, which is strongly connected to the population expansion history in the Pleistocene and Pliocene (Bell 2000), had a central contribution to the species’ current genetic structure.

To better underline this situation, we also indicate that in our dataset, 12 out of the 14 species with geographically distinct genetic lineages are resident, which brings further evidence that movement patterns plays an important role in shaping genetic diversity. Our results, in conjunction with previous research, indicate that most of the migrants from warm areas are genetically mixed, while species better adapted to cold climates show less admixture. This implies that species like the Hazel Grouse, Western Capercaillie, and ptarmigans could have spent some of the ice ages in small mountain refugia, inside the ice sheet (Lagerholm et al. 2017) (see Fig. 4). From a different perspective, some steppe species (Garcia et al. 2011) had their maximum distribution during the glaciations, when much of today’s central and Southern Europe had scarce vegetation and resembled a steppe formation (Frenzel et al. 1992). Similarly, the genus *Prunella* has been shown to have colonized new areas mainly during the glacial periods (Liu et al. 2017). Regarding the migratory movements of birds in the past 50,000 years, a recent study proposed that Old World species had relatively short movements (Somveille et al. 2020). In comparison, the same simulation-based study shows that birds in the New World were already doing transcontinental migrations. Overall, this brings further evidence that, in the past thousands of years, migratory behavior had an important role in shaping today’s genetic background of bird species. Additional factors like the time of glacial isolation, habitat selection, variation of the ecological niche through time (Eyres et al. 2021), geographical barriers and hybridization have also been crucial in species’ phylogeographical structure (Avise 2000).

In the dataset we compiled, several bird species show two main mtDNA haploclades. The Eurasian Collared Dove (Bagi et al. 2018), Common Redstart (Hogner et al. 2012) and Red-backed Shrike (Pârâu et al. 2019) are characterized by two main haplotype clades, with no geographic structure. However, the Little Owl (Pellegrino et al. 2014) and Great Reed Warbler (Hansson et al. 2008), also with two main mtDNA groups, display Western and Eastern European specific haploclades. In addition, species like the Skylark (Zink et al. 2008) and Great Grey Shrike (Olsson et al. 2010) which are spread across the entire Palearctic also show two divergent clades: Western Palearctic versus East Asian individuals. Previous research on the North American continent has revealed a similar situation for the Snow Goose (Quinn 1992) and Common Raven (Webb et al. 2011), which show two haploclades, with a relatively clear geographic structure. The most plausible explanation for the occurrence of two or more haploclades are the glacial refugia (Weiss and Ferrand 2007). The thousand years spent in these Southern refugia e.g. Iberian Peninsula or the Balkans in Europe, have acted as a selective force on genetic lineages. During the cyclical back-and-forth population expansion processes associated with ice ages, only certain populations survived, became in contact, and interbred. Although in the past 12 thousand years there was no ice age in the Western Palearctic and birds from different refugia freely mixed, the genetic legacy of the cold ages is still deeply rooted in the DNA. Furthermore, several species still have refugia-specific haplotypes, like in the case of the Rook (*Corvus frugilegus*) (Salinas et al. 2021).

However, in terms of species with several haploclades or sister species sharing haplotypes, hybridization and introgression of gene flow also represent a valuable explanation. Avian hybridization has long been a point of interest for ornithologists and geneticists, as evidence for active speciation, or in simple terms—seeing in real-time how a species is borne (McCarthy 2006; Ottenburghs et al. 2015). Hybridization is known to occur in 9% of the bird species (Grant and Grant 1992). In these cases, genetic material from one species is incorporated into another, potentially enforcing speciation (Rheindt and Edwards 2011; Ottenburghs et al. 2017). In Europe, the classic example is the Italian Sparrow (*Passer italiae)*, which still shares mtDNA haplotypes with its parent species, the Spanish Sparrow (*Passer hispaniolensis*) and the House Sparrow (*Passer domesticus*), representing an admixture of both genetic and phenotypic factors (Hermansen et al. 2011; Trier et al. 2014; Sætre et al. 2017). The dawn of the genomic era already provides strong molecular evidence into the complexity of genomic regions directly responsible for speciation, and promising a much better understanding in the years to come (Joseph 2018).

The Western Palearctic also includes a series of oceanic islands situated at low latitudes i.e. Macaronesia, which experienced different climate conditions during the Pliocene and Pleistocene (Webb and Bartlein 1992). These oceanic islands, which were not connected by land bridges with the continent were not affected by glaciations, which represents the triggering factor for genetic admixture in the avifauna of continental Western Palearctic (Wink 2018a). As a result, bird populations developed independently on these islands for several million years and acquired distinct genetic lineages (Avise and Walker 1998). Species like the Common Chaffinch (Rodrigues et al. 2014b), Common Blackbird (Rodrigues et al. 2016), European Robin (Dietzen et al. 2003, 2015; Rodrigues et al. 2013) and the Goldcrest (Rodrigues et al. 2014a) show island or archipelago unique haplotypes across Macaronesia, which are not shared with the continental conspecific population. Furthermore, due to recent molecular studies, island taxa which were previously considered subspecies have been elevated to species level: the Cape Verde Shearwater (Gómez-Díaz et al. 2006) and both the Tenerife and Gran Canaria Blue Chaffinches (Lifjeld et al. 2016; Sangster et al. 2016). Taking into consideration that the current taxonomic trend is to split species which have geographically distinct lineages, we expect to reach an increased number of approximately 18 thousand bird species (Barrowclough et al. 2016), from the 11 thousand currently recognized.

This circumstance of continental bird species being characterized by genetic admixture does not represent a special feature of the Western Palearctic avifauna, but rather the general situation for birds in the Northern temperate areas. Research on the North American continent has revealed similar patterns in several bird species (Zink 1996; Avise and Walker 1998; Dohms 2016), with most populations sharing haplotypes and only a handful of examples for geographically distinct genetic lineages.

For avian species in areas not affected by glaciations in the past few million years e.g. tropical areas and oceanic islands (see above), genetic structure and differentiation is, in many species, distinct. For example, the gnatcatchers and gnatwrens (Polioptilidae), pectoral sparrows (*Arremon taciturnus*), tyrant-manakins (Pipridae) and the Straight-billed Hermit (*Phaethornis bourcieri*) from South America show very distinct genetic groups, with almost no gene flow (Araújo-Silva et al. 2017; Capurucho et al. 2018; de Melo et al. 2018,2020; Smith et al. 2018). A similar situation has been described for the Wedge-billed Woodcreeper (*Glyphorynchus spirurus*) (Fernandes et al. 2013), the Southern Chestnut-tailed Antbird (*Sciaphylax hemimelaena*) (Fernandes et al. 2012), both Spotted and Spot-backed Antbirds (*Hylophylax naevioides/ naevius*) (Fernandes et al. 2014) and lowland antpittas (Grallariidae) (Carneiro et al. 2018). The consensus of the above-mentioned research is that genetic diversification in South American bird species was mainly triggered by the consolidation of Amazonian rivers and drainage system, which acted as dispersal barriers (Haffer 1969; Silva et al. 2019). These geological events took place during the Miocene and Pliocene (Rull 2011), which gave several million years of additional speciation for neotropical birds, in comparison to the birds from the Western Palearctic. Furthermore, the South American continent had a less fluctuating climate during these eras, a crucial factor contributing to species delimitation. A recent study indicated similar effects of the major geological events in the past million years on local birds phylogeography in Australia (Dolman and Joseph 2015). For the African continent, both river barriers and large vegetation shifts promoted bird speciation (Voelker et al. 2010, 2013).

In regard to other fauna and flora taxa inhabiting the Western Palearctic, certain degrees of genetic variation can be observed and very often, individuals can be linked to certain populations or geographic areas. This is illustrated by recent studies on Brown Hares (*Lepus europaeus*) (Minoudi et al. 2018), Stone Martins (*Martes foina*) (Tsoupas et al. 2019), plus Balkan Mole (*Talpa stankovici*) and European Mole (*Talpa europaea*) (Tryfonopoulos et al. 2010), which revealed that populations from the Balkans have region specific haplotypes. However, the Wild Cat (*Felis silvestris*) only shows five main geographic groups across the whole of Europe, with some populations hybridizing with domestic cats (Mattucci et al. 2016). Similarly, the European Roe Deers (*Capreolus capreolus*) and European Wild Boars (*Sus scrofa*) are characterized by a three clade pattern (Scandura et al. 2008; Sommer et al. 2009). Overall, the carnivores tend to exhibit region specific lineages, as has been shown in the Golden Jackals (*Canis aureus*) (Rutkowski et al. 2015), the Brown Bears (*Ursus arctos*) (Swenson et al. 2011) and the Grey Wolves (*Canis lupus*) (Pilot et al. 2010). Smaller mammals, such as the Field Vole (*Microtus agrestis*) and the Wood Mouse (*Apodemus sylvaticus*) equally show geographically specific clades across Europe (Jaarola and Searle 2002; Michaux et al. 2003). Reptiles and amphibians, which show very limited mobility, are defined by even more distinct genetic lineages (Joger et al. 2010), as indicated by the Tree Frogs (*Hyla arborea*) (Dufresnes et al. 2019), the Blotched Snakes (*Elaphe sauromates*) (Jablonski et al. 2019)*,* the Grass Snakes *(Natrix natrix)* (Kindler et al. 2017) and the Ocellated Skinks (*Chalcides ocellatus*) (Kornilios et al. 2010), to name just a few. Several studies in plants have also revealed comparable genetic differentiation (see ivy *Hedera sp.* (Grivet and Petit 2002) and numerous tree species (Petit et al. 2005)). One study based on mtDNA indicates that the European Stag Beetle (*Lucanus cervus*) has two main lineages, one restricted to the Balkan peninsula, while the second one is widely distributed in Europe (Cox et al. 2019). For the European Stone Crayfish (*Austropotamobius torrentium*), several region specific lineages were also uncovered by sequencing the mtDNA (Pârvulescu et al. 2019).

The above examples offer evidence that a species’ phylogeographic status is shaped by a combination of factors, including its locomotive capacities, fidelity to both breeding and migratory areas, as well as the age of the respective species. Furthermore, the current genetic population structure of a species is just a temporary step in its evolution (Avise 2000). To illustrate, species that are characterized by panmixia in present might be undergoing an active process of speciation and lineage sorting, such as the Great Tit (*Parus major*), which has been shown to have differentiating genomic elements in peripheral populations (Spurgin et al. 2019).

To conclude, genetic admixture in Western Palearctic birds (except the birds from the Macaronesia islands) represents a result of past climatic events, which occurred during the Pleistocene and Pliocene, as well as the high vagility of birds (unparalleled by other taxa), which helped to achieve such high gene flow.

### The choice of molecular markers

To date, the majority (i.e. 132 out of 198) of avian phylogeographic studies have employed nucleotide sequences of mtDNA as a molecular marker. In avian and other taxa population history, mtDNA has been a pivotal method which helped the field of phylogeography flourish (Avise 2004; Beheregaray 2008). It has been primarily used in initial surveys of population demography and biography, due to its low cost and efficiency (Mindell 1997). However, mtDNA does have limitations, as it is non-recombinant and maternally inherited (Krebs et al. 2018). These shortcomings have ignited a number of debates (Hebert et al. 2003; Ballard and Whitlock 2004; Hurst and Jiggins 2005; Edwards and Bensch 2009), but we consider that the advantages easily overcome the drawbacks (Rubinoff and Holland 2005; Sequeira et al. 2008; Zink and Barrowclough 2008). To bring further support for mtDNA, we argue that in our dataset, two different studies on the Saker Falcon (*Falco cherrug*), one employing mtDNA (Nittinger et al. 2007) and the more recent one, using SNPs (Zhan et al. 2015), produced the same phylogeography for the species. Similarly, two studies on the Eurasian Curlew (*Numenius arquata*), one using nuclear and mitochondrial DNA (Rodrigues et al. 2019), and one with NGS (Tan et al. 2019), yielded comparable results.

After mtDNA, microsatellites and nuDNA are the most commonly used markers. With the accompanying benefits of these two last markers (Avise 2004), the main restrain is that the focus is on a small strain of DNA.

Concerning the low scale usage of NGS (seven out of 198) across the studies included in our review, this is a relatively novel technology (see Fig. 1) and we argue that the costs still represent an impediment for many research groups. Although the sequencing costs are dropping fast, harnessing and affording high-quality computational analyses represents the major drawback. Unfortunately, bioinformatic expert support is still a luxury for many bird research labs.

As a final regard, we envision mtDNA will continue to provide robust first phylogeographic assessments for many years to come and we expect an increase in studies based on NGS data.

### What the future holds?

Until present, the bulk of animal phylogeography research has been dominated by mtDNA (Emerson and Hewitt 2005; Avise et al. 2016). With the advent of sequencing techniques, coupled with a decrease in running costs, we expect that the era of big “omics” data will revolutionize the phylogeographic research. However, regarding the later costs, the expenses for computational analyses, computer clusters and human bioinformatic resources are increasing (Muir et al. 2016). Whole-genome sequencing will challenge the present image offered by mtDNA (Kraus and Wink 2015; Ottenburghs et al. 2019) and we anticipate that a number of species currently characterized by panmixia will reveal a certain degree of differentiation. Furthermore, the maturation of phylogeography will benefit not only from developments in DNA sequencing techniques, but also from the advancement of theory and statistical analyses in this field (Stiller and Zhang 2019).

Finally, bird populations are very dynamic and their distribution in space and time are affected by multiple factors such as climate, availability of habitat and food but also, in the past hundred years, anthropogenic threats. This complex network of factors has a fundamental influence on shaping their future phylogeography (Kumar and Kumar 2018). With climate change, unprecedented human-driven alteration of the environment and overall decrease of food stocks (e.g. insects), genetic consequences on bird populations might not be that far away.

## Conclusions

After 30 years of studies in phylogeography, we have a good understanding of avian population history in the Western Palearctic, based on the 145 species whose genetic background has been elucidated. The majority of them show high levels of genetic admixture, whereas the species inhabiting the oceanic islands (i.e. Macaronesia) are resident and developed distinct genetic lineages. The panmixia is the legacy of the Pleistocene and Pliocene climatic fluctuations, which forced the birds to cyclically retreat in refugia only to subsequently expand and recolonize higher latitudes, after the ice sheet retreat. These events caused population admixture, both in the refugia and at the contact zones, during population expansion. The bulk of the avian phylogeographic information comes from nucleotide sequences of mtDNA, which, with few limitations, has proven to be a robust and trustworthy molecular marker. With the current dawn of big genomic data in bird research, which offers a much higher resolution than previous studies, we envision a steep increase of NGS-driven phylogeography studies. These new studies have both the power to offer initial population structure surveys and, most important, to challenge previous views based on other markers. Nonetheless, the utility of mtDNA when used with due understanding and in conjunction with NGS is clearly very high. We, therefore, urge our peers not to forget mtDNA completely, which has been a great companion in the past 30 years.

## Supplementary Information

Below is the link to the electronic supplementary material.

Supplementary file1 (XLSX 29 KB)

Supplementary file2 (XLSX 26 KB)

Supplementary file3 (PDF 3272 KB)

Supplementary file4 (XLSX 16 KB)
