# Supplementary material for: Common patterns in the molecular phylogeography of western palearctic birds: a comprehensive review
Source: J Ornithol. 2021 May 13;162(4):937–59. doi: 10.1007/s10336-021-01893-x (PMC8118378; doi:10.1007/s10336-021-01893-x)
Supplement: Supplementary file 3 — Supplementary file3 (PDF 3272 KB) [file 10336_2021_1893_MOESM3_ESM.pdf]

Figure S3: Comparative haplotype networks for the three species of birds, representative for each phylogeographic category: a) European Turtle Dove (*Streptopelia turtur*) indicating panmixia, b) European Green Woodpecker (*Picus viridis*) showing low geographic differentiation and c) African Blue Tit (*Cyanistes teneriffae*) indicating geographically distinct lineages.

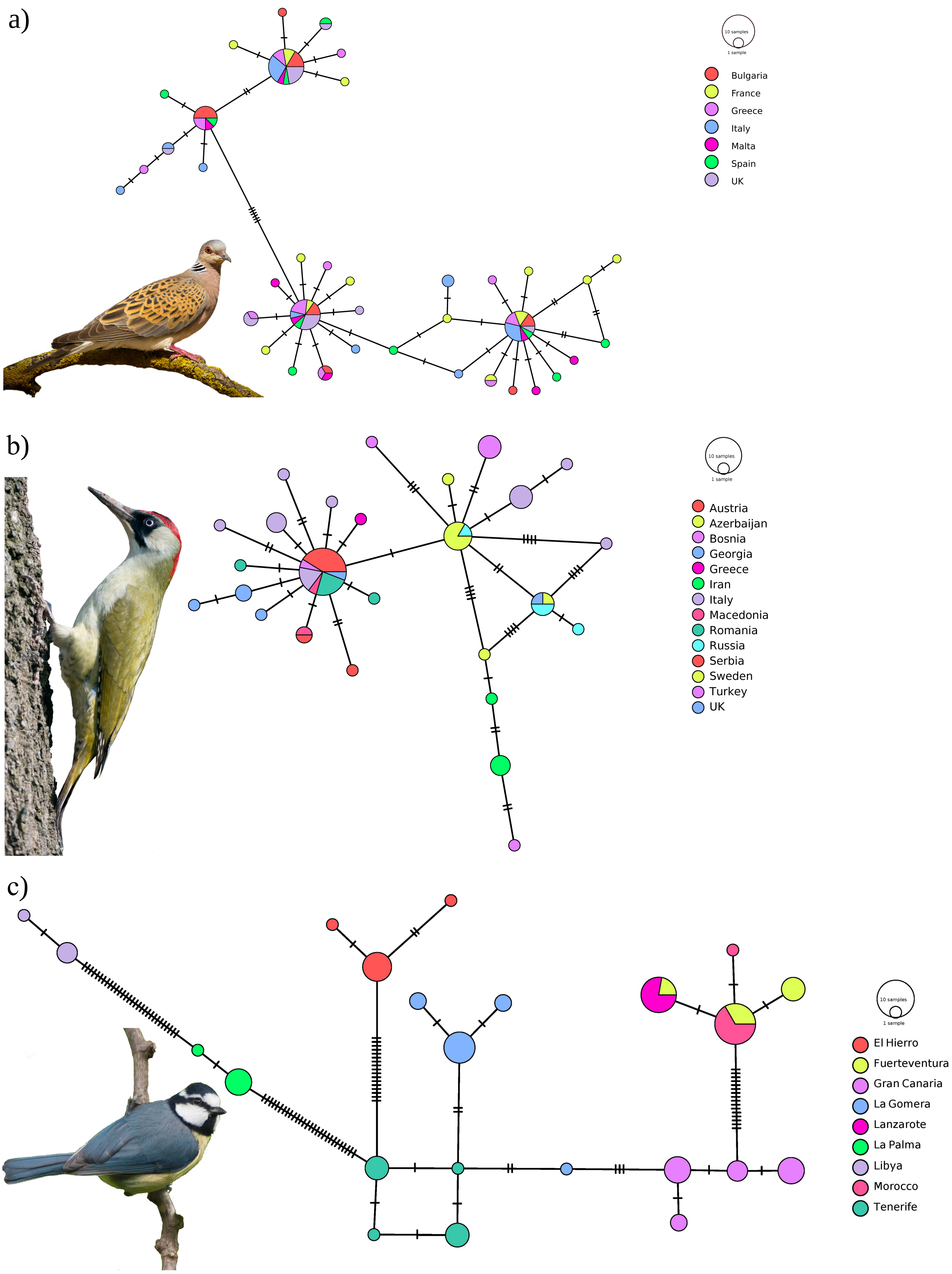

a)
10 samples
1 sample
Bulgaria
France
Greece
Italy
Malta
Spain
UK
b)
10 samples
1 sample
Austria
Azerbaijan
Bosnia
Georgia
Greece
Iran
Italy
Macedonia
Romania
Russia
Serbia
Sweden
Turkey
UK
c)
10 samples
1 sample
El Hierro
Fuerteventura
Gran Canaria
La Gomera
Lanzarote
La Palma
Libya
Morocco
Tenerife
